# The risk of osteopenia/osteoporosis and psoriatic disease: A systematic review

**DOI:** 10.1002/ski2.169

**Published:** 2022-09-21

**Authors:** Anna Schauer, Aarthy K. Uthayakumar, Glenn Boardman, Christopher B. Bunker

**Affiliations:** ^1^ Dermatology Department University College London Hospitals NHS Trust London UK; ^2^ Research Department Fiona Stanley Hospital Perth Western Australia Australia

## Abstract

**Background:**

Psoriasis (Ps) is a multisystem inflammatory disease associated with several comorbidities; however, its effect on bone health remains uncertain. This systematic review aimed to evaluate the risks of osteopenia (OPe) and osteoporosis (OP) in psoriasis.

**Methods:**

A systematic search was performed for published studies evaluating cutaneous Ps and psoriatic arthritis (PsA) compared with healthy control groups utilizing a validated bone mineral density (BMD) assessment score. Meta‐analysis was performed using a random‐effects model; pooled estimates and their confidence intervals (CIs) were calculated. For analysis, Ps and PsA groups were combined due to the small number of studies.

**Results:**

Twenty‐one studies were included for final analysis; three Ps only, 15 PsA and three both. There was a significant difference between psoriatic disease (combination Ps and PsA group) compared with controls relating to an association with OP/OPe, with an overall odds ratio (OR) of 1.71 (95% CI 1.07–2.74: *p*‐value = 0.026). The Ps group had significantly lower BMD than the control group at both the lumbar spine and femoral neck (mean difference −0.04; 95% CI −0.090 to 0.002 and −0.03; 95% CI −0.059 to 0.003 respectively).

**Conclusion:**

Putative risks of OPe and OP in both Ps and PsA are supported but not confirmed. Significant heterogeneity of reported data limits definitive conclusions in this meta‐analysis. This review contributes to the further understanding of Ps as a multisystem disease and future management of potential comorbidities, but highlights key gaps in the literature. Further studies addressing standardised OP reporting, specific disease group characteristics comparing Ps with PsA, patient characteristics and medication use, are required in order to make more certain conclusions with greater clinical impact.

## Graphical abstract

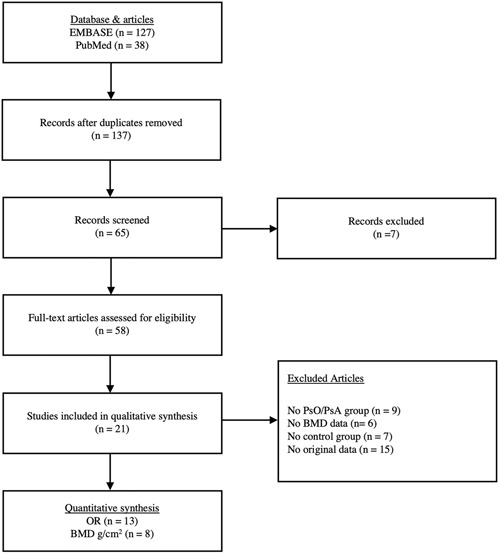

This systematic review evaluates the risks of osteopenia and osteoporosis in psoriasis. We exclusively included studies with healthy control comparison groups and which used outcomes of validated bone quality assessment measures. Meta‐analysis was performed using a random‐effects model and pooled estimates and confidence intervals calculated. This review contributes to the further understanding of Ps as a multisystem disease and future management of potential comorbidities, but highlights key gaps in the literature. Further studies addressing standardized OP reporting, specific disease group characteristics comparing Ps with PsA, patient characteristics and medication use, are required in order to make more certain conclusions with greater clinical impact.

1

**What is already known about this topic?**

- Psoriasis (Ps) and osteoporosis (OP) are both chronic inflammatory conditions with complex pathogenesis.
- Several patients with psoriasis and psoriatic arthritis have risk factors for osteoporosis; long term topical corticosteroid use, treatments impacting bone remodelling including non‐steroidal anti‐inflammatory drugs, and lifestyle factors.
- Current guidelines on managing comorbidities of psoriasis do not address bone health and previous studies evaluating the association between Ps and OP have produced conflicting results.

**What does this study add?**

- This systematic review is the first evaluating bone mineral density (BMD) and psoriatic disease exclusively in studies with healthy control comparison groups and using outcomes of validated bone quality assessment measures. We demonstrate the possible risks of osteopenia and osteoporosis in psoriatic disease.
- Given inconsistencies, bias, and heterogeneity of reporting in the existing published literature, more extensive longitudinal studies with standardized BMD reporting measures and subgroup analyses, are needed to better characterize the risk of low BMD in psoriasis and medication risk factors.

## 2 INTRODUCTION

Psoriasis (Ps) is a chronic inflammatory disease affecting the skin and joints, and is increasingly associated with comorbidities including obesity, insulin resistance, cardiovascular disease, depression, dyslipidaemia, and arrhythmias.^1^, ^2^ Osteoporosis (OP) represents a disruption in bone microarchitecture and low bone mass, reducing bone strength and increasing fracture risk. OP is diagnosed using bone mineral density (BMD) assessment by dual‐energy X‐ray absorptiometry (DXA), with a *T* score ≤−2.5 and between −1.0 and −2.5 standard deviations (SD), indicating OP and osteopenia (OPe) respectively.^3^, ^4^

Chronic inflammation is a vital component in OP pathogenesis. OP and Ps have many overlapping pathways, including raised systemic inflammatory cytokines, namely interleukin‐6, interferon‐γ and tumour necrosis factor‐α.^1^, ^2^ Further, psoriatic arthritis (PsA) shares a similar *T* helper type 1 and 17 driven inflammatory mechanism with rheumatoid arthritis. Rheumatoid arthritis is known to be associated with OP, suggesting that Ps may also potentially be associated with OP.^5^

In addition to sharing molecular similarities, there are clear potential risk factors for OP in patients with psoriatic disease. Physical inactivity, compounded by arthralgia or joint dysfunction, and lifestyle factors including smoking or alcohol may contribute.^1^ Systemic corticosteroid treatment is a known risk factor for OP. Topical corticosteroids (TCS) are suggested to be an independent risk factor for OP, particularly the use of potent or very potent TCS in high cumulative doses.^6^ Clinical guidelines for Ps recommend the long‐term use of TCS, potentially involving application over large surface areas for prolonged periods. Other Ps treatments may also affect bone remodelling, including non‐steroidal anti‐inflammatory drugs (NSAIDs), disease‐modifying anti‐rheumatic drugs (DMARDs) including methotrexate (MTX), azathioprine (AZA) and ciclosporin (CsA), or biologic agents.

Current guidelines on managing comorbidities in Ps do not address bone health. Published studies evaluating the association between Ps and OP have produced conflicting results. This systematic review aimed to evaluate the risks of OPe/OP in Ps and PsA patients to better guide management.

## 3 METHODS

### 3.1 Search strategy and selection criteria

This systematic review was conducted according to the Preferred Reporting Items for Systematic Reviews and Meta‐analyses (PRISMA) statement.^7^ The electronic databases PubMed and Embase were screened from their inception through to 31 May 2021, using the keywords “*psoriasis*” OR “*psoriatic*” AND “*osteoporosis*” OR “*osteopenia*” OR “*bone*” OR “*fracture*”, to identify studies evaluating associations between Ps and OP. The search was restricted to human subjects, publications in English and controlled trials. A detailed search strategy of electronic databases can be found in Tables S1 and S2. References of included studies were reviewed, as were systematic reviews on similar topics.

Studies that satisfied the following criteria were eligible for inclusion: case‐control, cohort, or cross‐sectional studies with original data and more than 10 patients; study population with Ps or PsA; control groups without psoriatic disease; and reported outcomes of effect estimates of OPe/OP or BMD *T*‐score. Studies not reporting the primary outcome were excluded.

### 3.2 Data extraction and outcomes

Search results were compiled into a single Endnote file, with duplicates removed. Two investigators screened titles and abstracts (AS,AKU) independently, with discrepancies resolved by a third‐party reviewer (CBB), to identify articles for full qualitative review. If the same dataset appeared in different reports, the earliest study was included.

The primary outcome of this systematic review was to define the association between psoriatic disease and OPe/OP. The main outcomes of interest were the incidence and prevalence data of OPe and OP and absolute values of BMD as an ancillary marker. Secondary outcomes included data on the effect of concurrent psoriatic treatments.

### 3.3 Assessment of bias

Bias was assessed for each study included in the systematic review by two independent reviewers (AS,AKU). A modified Newcastle Ottawa Scale (NOS) was applied to each study to assess for bias in the selection and comparability of study groups, and ascertainment of outcomes of interest^8^ (Tables S3–S5). A score of seven or higher was considered high quality.

### 3.4 Data synthesis and statistical analysis

The prevalence/incidence data, and BMD for the psoriatic disease cohort and control group were presented. The estimated odds ratios (OR) and 95% confidence intervals (CI) were calculated for the primary binary outcomes (risk estimates of OPe/OP). For continuous outcomes (absolute value of BMD) the mean differences (MD) were calculated. Due to the heterogeneity in *T*‐score reporting, quantitative analysis was not possible. Given the small number of studies evaluating Ps alone in comparison to a combination of Ps and PsA, separate subgroup analysis was not performed due to the variability of small groups.

Pooled prevalence estimates (expressed as OR) and BMD across studies were generated using a random‐effects model, and heterogeneity between studies was evaluated using a *χ* ^2^ test on Cochran's Q quantified with the *I* ^2^ statistic. A *p*‐value of <0.05 was used as a cut‐off to indicate significant heterogeneity, whereas, on *I* ^2^ statistic, limits of 30%, 50% and 75% indicated low, medium and high levels of heterogeneity, respectively.^9^ A random‐effects framework was utilized given the variation in population and characteristics across the studies. Meta‐analysis was performed with R version 4.1.0 and package Meta generating a forest plot showing fixed and random‐effects models, using Mantel‐Haenszel for OR and inverse variance for BMD. Inter‐study heterogeneity was assessed using DerSimonian and Laird variance estimator and evaluated using a *χ* ^2^ test on Cochran's Q test and quantified with the *I* ^2^ statistic.

### 3.5 Effect measures

Publication bias was assessed with the aid of funnel plots. Influence analysis was performed to evaluate the impact of each study on the overall effect by omitting them individually. The publication bias was quantified using Eggers's test; this is considered statistically significant when *p* < 0.05.

## 4 RESULTS

**FIGURE 1:**
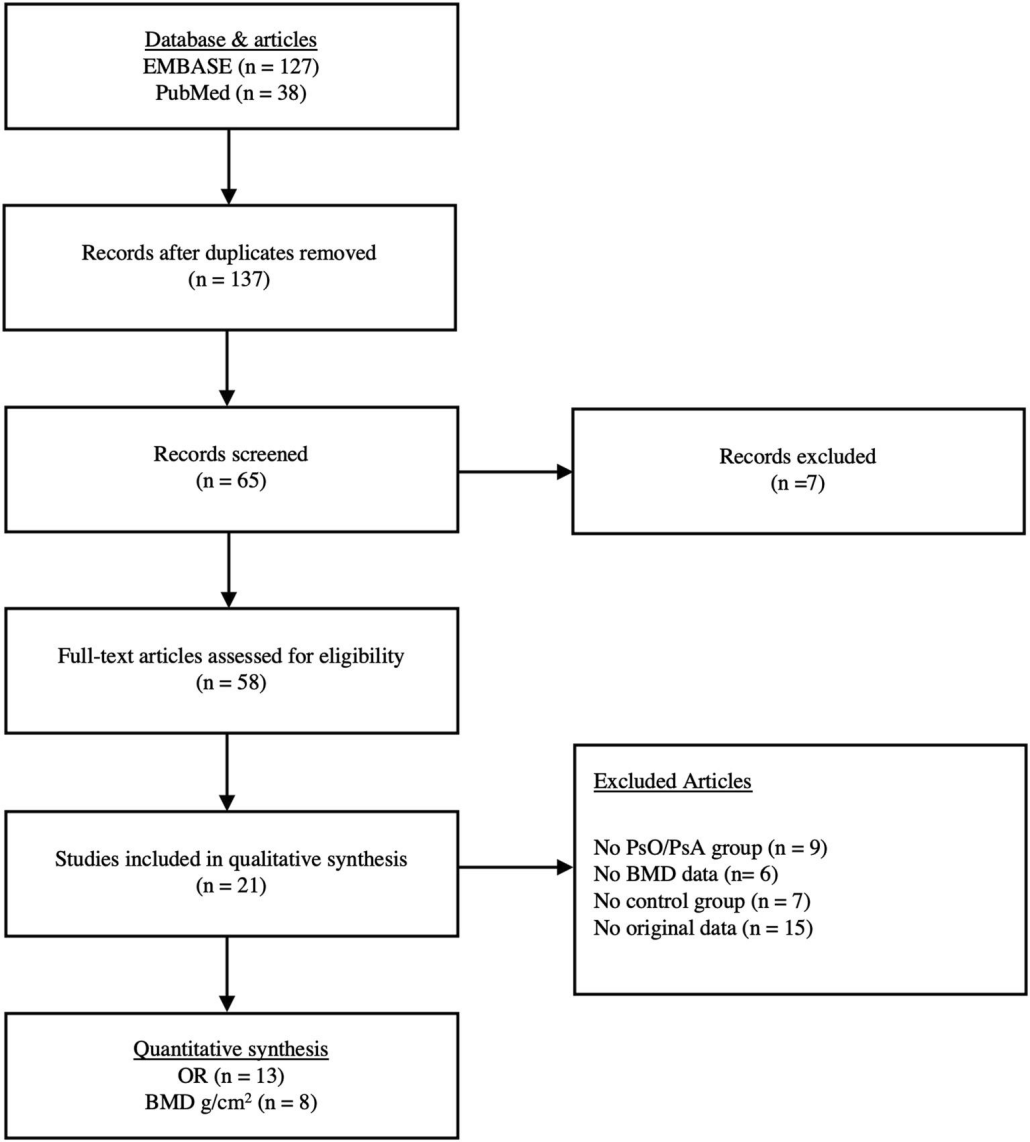
Prisma protocol for systematic review

From an initial selection of 165 references, 79 were excluded after reading the title or abstract as duplicates or irrelevant, and 37 after reading the article; 21 articles were retained. Screening references did not yield any additional studies. The selection process is further detailed in Figure 1.

### 4.1 Study population and characteristics

Overall, 21 studies (Table 1) compared Ps patients with a control group, specifically Ps (*n* = 3), PsA (*n* = 15) and both, as separate comparison groups (*n* = 3). Hereon ‘psoriatic disease’ refers to Ps and PsA groups combined. Two further studies included additional comparison groups: pre‐ and post‐menopausal females, and men. In all studies, the diagnosis of Ps was made clinically in the Dermatology outpatient setting, and the diagnosis of PsA predominantly by Classification criteria for Psoriatic Arthritis (CASPAR) diagnostic criteria or the International Classification of Diseases (ICD) for large national databases. Cases were enroled from various settings (specialist Dermatology/Rheumatology outpatient [*n* = 11], inpatient Dermatology ward [*n* = 1], large national databases [*n* = 6]), with sample sizes reflecting this and a total of 267 415 overall cases included. Control groups comprised of healthy individuals and were primarily age and gender‐matched (*n* = 15). The modified NOS appraisal scale deemed study quality as high 7 (*n* = 5); intermediate 4–6 (*n* = 14) and low 3 points (*n* = 2); noting that cross‐sectional studies may score a maximum of 6 points (*n* = 13).

**TABLE 1:** Summary of included studies

				Exclusion criteria	Age^a^	NOS^e^ (total score)
					Female^b^	
					Menopause^c^	
Study	Design	Number of participants & details			BMI^d^	
Attia, 2011, Egypt^10^	Case control	34 Ps	Derm/Rheum OPC (CASPAR)	Postmenopausal females; smoking history; alcohol abuse, systemic corticosteroids <12 months	36	S**
					36	C**
		16 PsA			0	O***
					NA	(7/9)
		20 controls	Age, sex matched, healthy, no FHx Ps			
Botticella, 2013, Italy^11^	Cross‐sectional	65 PsA	Subgroups +/− bone erosions on hand & feet x‐ray	NA	61.4	S*
					NA	C*
		30 controls	Age matched, healthy		NA	O*
					NA	(3/6)
Cortet, 2002, France^12^	Cross‐sectional	24 PsA	Hospital OPC (Wright & Moll)	Current medications affecting bone metabolism (including corticosteroids)	50.2	S***
					50	C**
		48 controls	Age, sex, attending OPC for disorders unrelated to bone		25	O*
					25.6	(6/6)
Dheda, 2003, South Africa^13^	Cross‐sectional	20 PsA	Indian patients, subgroups ≤9/≥9 years disease duration	>55 yoa (post‐menopausal)	38.2, 40.3	S*
					35	C**
					100	O*
		20 controls	Age, sex matched		NA	(4/6)
Dreiher, 2009, Israel^1^	Case control	7936 Ps	Dataset Clalit health services	<51 yoa	65.6	S****
		14 835 controls	Age, sex matched		48.2	C**
					100	O**
					NA	(8/9)
Haddad, 2017, Israel^14^	Cross‐sectional	3161 PsA	Dataset Clalit health services 2002–2014	NA	58.4	S***
					53.4	C**
					NA	O*
		31 620 controls	Age, sex matched, no rheumatic disease/Ps			
					27.5	6/6
Kaine, 2019, US^15^	Retrospective cohort	14 898 PsA	National claims database 2008–2015 (ICD)	Continuous enrolment with medical/pharmacy coverage <24 months prior to & <12 months post index date	53.4	S****
					55.4	C**
		35 037 controls	Age, sex, residence, index date matched, no PsA		NA	O***
					NA	9/9
Kathuria, 2017, US^16^	Cross‐sectional	183 725 Ps	National ED attendance 2006–2012 (ICD)	NA	54.4, 56.1	S***
		28 765 PsA			48.5, 55.2	C
		197 918 710	Numbers Ps/PsA attending ED		NA	O*
		198 073 760 controls			NA	4/6
Krajewska‐Wlodarczyk, 2017, Poland^17^	Cross‐sectional	51 PsA	Rheum/Derm OPC (CASPAR)	Pre‐menopausal; systemic corticosteroid (current/<2 years); thyroid disease; pacemaker; joint implant	65.6	S**
					100	C
					100	O*
		44 controls	Females, no inflammatory joint disease		30.1	3/6
Kreeshan, 2014, UK^18^	Case control	56 PsA	Referred to hospital for BMD assessment	NA	59.5	S**
					56	C**
		56 controls	Age, sex matched, BMD referral with no indication		100	O**
					NA	6/9
Lee, 2020, Korea^19^	Retrospective cohort	25 306 Ps	Korean National Health Insurance screening service (ICD)	History of OP	Banded	S****
					50.8	C**
					NA	O***
		101 224 controls	Age, sex, income, residence, index date, similar mean follow‐up matched, no Ps			
					Banded	9/9
Lo Giudice, 2020, Argentina^20^	Retrospective cohort	22 PsA	Rheum OPC 2000–2017 (CASPAR)	Fragility fracture prior to PsA diagnosis; <1 year follow‐up	50.2	S****
					47.8	C**
					NA	O***
		46 controls	Age, sex matched		NA	9/9
Martinez‐Lopez, 2018, Spain^21^	Cross‐sectional	58 PsA	Derm/Endo OPC, PASI>4, untreated OP	<18 yoa; <1 h outdoors/day; PsA, RA, IBD; previous phototherapy, systemic, biologic, calcium, vitamin D supplement	48.9	S**
		67 OPe/OP				
					43.1	C**
		61 controls	Residence matched		NA	O*
					30.3	5/6
Millard, 2001, UK^22^	Case control	20 PsA	Severe Ps, Derm inpatients	NA	47	S*
					50	C**
		20 controls	Age, BMI matched, 10 female volunteers UK adult Twin Registry, 10 male healthy staff		NA	O**
					26.6	5/9
Modalsli, 2016, Norway^23^	Cross‐sectional	2804 Ps	Population‐based database	<20 yoa; required information missing	Banded	S***
					52	C*
		45 390 controls	Age matched		NA	O*
					NA	5/6
Nolla, 2002, Spain^24^	Cross‐sectional	52 PsA	Rheum OPC, non‐axial PsA, subgroups 19 men, 14 premenopausal & 19 post‐menopausal female	Disease duration <1 year; current/past systemic corticosteroid therapy; other disease affecting bone metabolism; ankylosing spondylitis; inflammatory/degenerative hip involvement	51.9	S**
					63.4	C**
					37	O*
		52 controls	Age, sex, menopause status matched		NA	5/6
Pedreira, 2011, Brazil^25^	Cross‐sectional	52 Ps	Rheum OPC (CASPAR)	Pre‐menopausal; secondary OP/OA/sarcopenia; Steinbroker functional class IV; cognitive impairment; cancer/HIV; use of insulin, statins, GH, anabolic agents, HRT, TNF blockers, vitamin D	61.0	S**
		45 PsA				
					100	C**
					100	O*
					27.9	5/6
		98 controls	Healthy, female, age, sex, BMI, ethnic background, SES matched			
Petho, 2021, Hungary^26^	Cross‐sectional	118 PsA	Rheum OPC 2017–2018 (CASPAR)	Metabolic bone disease, malignancy, liver/kidney disease (control group)	54	S**
					56.8	C**
					NA	O*
		118 controls	Healthy, age, sex matched hospital volunteers/relatives		NA	5/6
Riesco, 2013, Spain^27^	Cross‐sectional	91 PsA	CASPAR, subgroups: 45 males, 15 premenopausal & 31 postmenopausal females	Radiological axial involvement; previous bilateral THR; physical/psychological impairment precluding study completion	54.0	S***
					50.5	C**
					34	O*
		91 controls	Age, sex, menopause status matched, no Ps, OP, inflammatory joint, autoimmune disease			
					28.6	6/6
Solak, 2016, Turkey^28^	Case control	43 Ps	No PsA, Derm OPC 2014–2015	<18/>50 yoa; systemic disease affecting BMD; phototherapy/systemic therapy for Ps in preceding 3 months	35.3	S**
					62.8	C*
		41 controls	Sex matched, healthy, hospital staff volunteers		0	O***
					24.9	6/9
Zhu, 2014, Hong Kong^29^	Cross‐sectional	53 PsA	Rheum OPC (CASPAR)	Other disorder affecting bone metabolism; pregnant/breastfeeding; use of AED, thyroid, anti‐osteoporotic therapy (corticosteroids, calcium & vitamins in cases only)	53.1	S**
					54.7	C**
		53 controls	Age, sex matched volunteers		48	O*
					25.2	5/6

*Note*: The stars refer to the Newcastle Ottowa Scale scoring on the criteria of Selection (S), Comparability (C), and Outcome (O) ‐ this is a standardised scoring tool used for systematic reviews.

Abbreviations: AED, anti‐epileptic drugs; BMD, bone mineral density; C, comparability of study groups; CASPAR, classification criteria for psoriatic arthritis; Derm, dermatology; ED, emergency department; FHx, family history; GH, growth hormone; HRT, hormone replacement therapy; IBD, inflammatory bowel disease; NA, not available; O, ascertainment of outcome of interest; OA, osteoarthritis; OP, osteoporosis; OPC, outpatient clinic; Ps, psoriasis; PsA, psoriatic arthritis; RA, rheumatoid arthritis; Rheum, rheumatology; S, selection of study groups; THR, total hip replacement; yoa, years of age.

^a^ Mean age (years).

^b^ Female sex (percentage).

^c^ Post‐menopausal (percentage).

^d^ Mean BMI (kg/m^2^).

^e^ NOS, Newcastle‐Ottawa Scale was scored with stars.

The mean participant age lay between 35 and 66 years; there was a female‐predominant sex distribution in most of the studies (*n* = 13) with two studies excluding males entirely. The mean BMI was between 24.9 and 30.3. Menopausal status was recorded (*n* = 10), excluded (*n* = 2) and otherwise not stated (*n* = 11).

### 4.2 Pooled effects on the primary outcome

**FIGURE 2:**
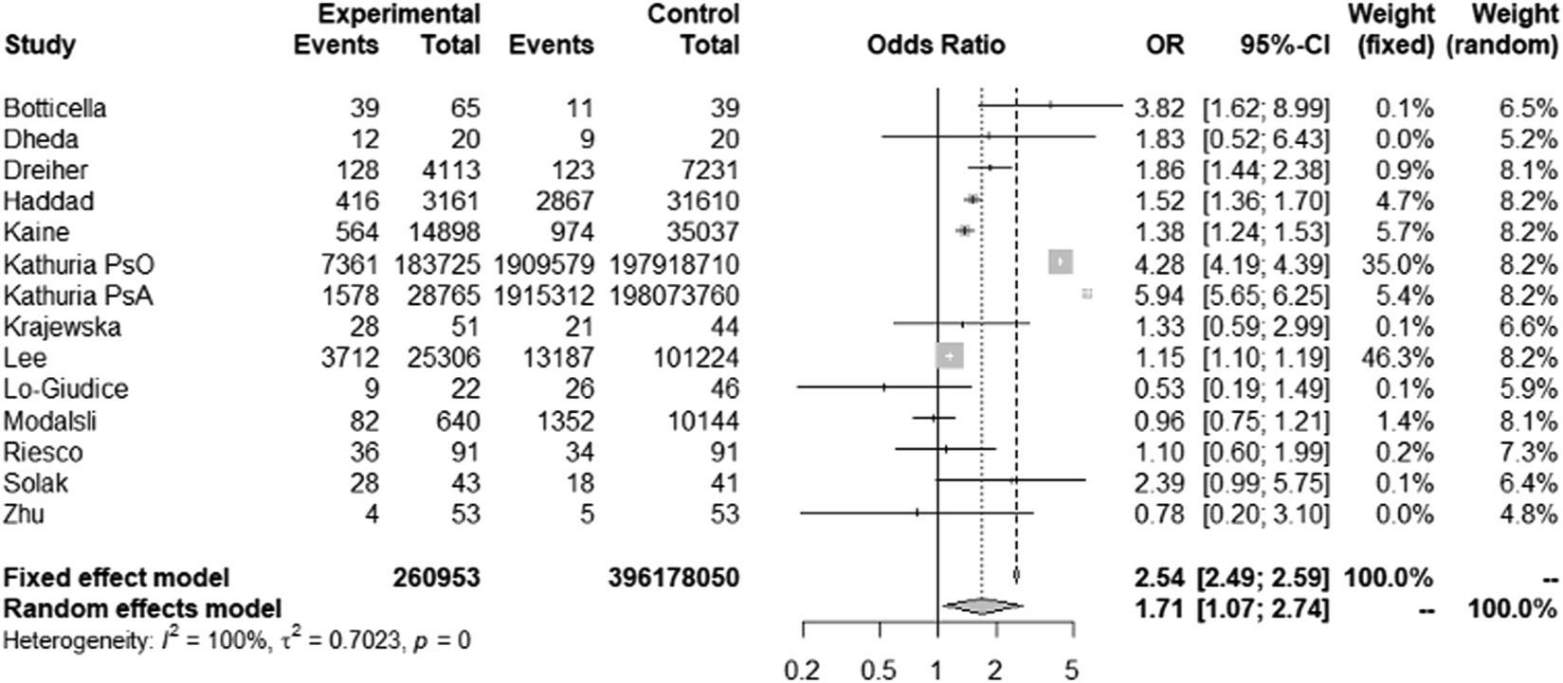
Pooled association between osteoporosis/osteopenia and psoriasis by measure of odds ratio

The association between OPe/OP and Ps using effect measures was reported in 14 studies, and these were pooled for meta‐analysis (Figure 2). The psoriatic disease group differed significantly from the control group, with an overall OR of 1.71 (95% CI 1.07–2.74: *p*‐value = 0.026). Only three studies did not show an increased risk of low BMD.

Sixteen studies (Table 2) reported BMD results using DXA scanning; nine reported absolute BMD (g/cm^2^) measures, subdivided into anatomical sites of the lumbar spine (LS) (*n* = 9), total hip (TH) (*n* = 4) and femoral neck (FN) (*n* = 7). The Ps group was found to have significantly lower BMD than the control group for both LS and FN (mean difference −0.04; 95% CI −0.090 to 0.002 and −0.03; 95% CI −0.059 to 0.003 respectively); but not TH (Figures S1–S3).

**TABLE 2:** Risk of OP/OPe by absolute BMD

Study	BMD measurement			Main significant outcome/s (controls)	Other significant outcome/s
	Device	Site	Outcome		
Attia^10^	DXA	LS, FN, DR	OPe/OP *T*‐score, *Z*‐score	Ps & PsA: Lower *T*‐score & *Z*‐score LS, FN, DR (*p* < 0.05)	Higher PASI associated with lower FN *Z*‐score
Botticella^11^	DXA	LS, TH	g/cm^2^, *T*‐score, *Z*‐score, OR	PsA: Lower BMD, *T*‐scores & *Z*‐score at LS & TH	Group A (with bone erosions) had lower BMD, T & Z scores at LS than group B (without bone erosions) (*p* < 0.005)
Cortet^12^	DXA, QUS	LS, FN, heel	g/cm^2^, *T*‐score, metabolic bone markers	PsA: Lower BMD at LS & FN	ICTP marker of bone turnover higher in PsA patients (4.81) versus controls (3.23) (*p* < 0.01)
Dheda^13^	DXA, Imaging	LS, FN	g/cm^2^, OR, number of damaged joints	No difference in mean BMD LS/FN PsA: *T*‐score <−1 in 60% (45%)	Correlation between number of damaged joints & degree of disability, with reduction in BMD; no correlation between BMD & duration of PsA
Dreiher^1^	NA	NA	OR	Ps: OP 12.4% (11.2%) [OR 1.12]	Ps: 56/109 chronic disease diagnoses more prevalent; more comorbidities (5.0 vs. 3.7); higher prevalence OP in males with Ps [OR 1.86] but not females
Haddad^14^	NA	NA	OR	PsA: Increased OP [OR 1.56]	PsA: Increased DM [OR 1.56] & hypothyroidism [OR 1.62]
Kaine^15^	NA	NA	OR	PsA: Increased OP [IRR 1.68 versus 0.94]	PsA: Higher prevalence of comorbidities
Kathuria^16^	NA	NA	OPe/OP, OR, #	Ps associated with OPe [OR 2.86] & OP [OR 2.97]; PsA associated with OPe [OR 4.13] & OP [OR 4.04]	Ps/PsA: Association with #
Krajewska‐Wlodarczyk^17^	DXA	LS, TH, FN	g/cm^2^, *T*‐score, *Z*‐score, OR	No difference in OP & OPe	PsA: Increased disease duration correlates with lower BMD FN & LS; increased sarcopenia 43.1% (20.4%) PsA with sarcopenia: OP 2× more common than without
Kreeshan^18^	DXA	LS, FN, TH	*T*‐score, OR	PsA: LS *T*‐score −1.4 (−0.65), FN *T*‐score −1.2 (−0.5); lower BMD [OR 1.03 LS, OR 1.68 TH]	Relationship remained adjusted for height & weight
Lee^19^	NA	NA	OPe/OP—adjusted HR & OR	Ps: OP in 14.7% (13%) [HR 1.09, OR 1.21]	Adjusted for <60/60+ yoa, gender, CCI score, obesity, smoking, alcohol, autoimmune disease, atopic dermatitis, psoriatic medication use; higher rates of comorbid inflammatory conditions for example, RA, atopic dermatitis
Lo Giudice^20^	DXA, imaging	NA	OR, #	PsA: OP 27.3% (26.1%); no difference in # incidence	NA
Martinez‐Lopez^21^	DXA	LS, TH	*T*‐score, metabolic bone markers	PsA: Lower *T*‐score LS & TH	Higher BMI associated with higher TH *T*‐score; no difference in # (vertebral, wrist, hip, pelvis); PsA: Lower vitamin D levels (*p* < 0.001)
Millard^22^	DXA, US	LS, TH	g/cm^2^, *T*‐score, *Z*‐score, OPe/OP	No difference in BMD	Adjusted for BMI/sex; PsA with psoriatic arthropathy: Lower mean LS *Z*‐scores −1.16 than those without arthropathy +1.38 (*p* = 0.015)
Modalsli^23^	DXA, imaging	LS, TH, FN	*T*‐score, OR, # (forearm, hip)	No association between Ps & *T*‐score or OP at these all sites [OR 0.77]; no increased # risk [HR 1.03]	NA
Nolla^24^	DXA	LS, FN	g/cm^2^, *T*‐score, *Z*‐score, OPe/OP	No difference in BMD	In post‐menopausal FN BMD lower (*p* < 0.05)
Pedreira^25^	DXA	LS, FN	g/cm^2^, FRAX	Ps/PsA: Higher #	Post‐menopausal PsA: Lower FN BMD; PsA: Recurrent falls & longer disease duration increased # risk [OR 18.3 & 1.08]
Petho^26^	DXA	LS, FN, DR	g/cm^2^, g/cm^3^, FRAX, metabolic bone markers	PsA: Lower areal & volumetric BMD	Age & years since menopause were determinant of BMD; volumetric BMD identified more patients with low BMD
Riesco^27^	DXA	LS, FN	g/cm^2^, *T*‐score, *Z*‐score, OR	No differences in BMD, *T*‐score & *Z*‐score	Increased disease duration associated with decreased BMD; PsA: Higher bone turnover, increased vitamin D deficiency; increased hip # overall & with longer disease duration, menopause, DMARD use, vitamin D deficiency
Solak^28^	DXA	LS, FN	*T*‐score, *Z*‐score, OR	Ps: Lower BMD (especially females); lower *Z*‐score LS & FN; higher OPe & OP	Ps: Higher BMI, increased # (especially postmenopausal females)
Zhu^29^	DXA, QCT	LS, TH, FN, DR	g/cm^2^, g/cm^3^, OR	PsA: BMD LS higher No difference FN, TH, DR or OP prevalence	PsA: Asymptomatic vertebral # more common (23.8 vs. 3.8%); cortical BMD lower (higher porosity); higher CRP & ESR

Abbreviations: #, fragility fractures; BMD, bone mineral density; BMI, body mass index; CCI, Charlson Comorbidity Index; CRP, C‐reactive protein; DM, diabetes mellitus; DR, distal radius; DXA, dual‐energy X‐ray absorptiometry; ESR, erythrocyte sediment rate; FN, femoral neck; FRAX, Fracture Risk Assessment Tool; HR, hazard ratio; ICTP, c‐terminal telopeptide; IRR, incidence rate ratio; LS, lumbar spine; NA, not available; OP, osteoporosis; OPe, osteopenia; OR, odds ratio; Ps, psoriasis; PsA, psoriatic arthritis; QCT, quantitative ultrasound; QUS, quantitative ultrasound; US, ultrasound.

The gold standard for reporting BMD is with *T*‐scores, and values were reported in 10 different studies, with specific measurements at LS (*n* = 9), TH (*n* = 3), FN (*n* = 8) (Table 3). Due to missing variables required for meta‐analysis, this was not performed.

**TABLE 3:** Risk of OP/OPe by DXA mean *T*‐score

	Ps/PsA			Control			Calculated difference		
	LS	TH	FN	LS	TH	FN	LS	TH	FN
Attia (Ps)^10^	−1.4	NA	−0.35	0.32	NA	0.54	−1.72	NA	−0.89
Attia (PsA)^10^	0.77	NA	−1.08	0.32	NA	0.54	0.45	NA	−1.62
Botticella^11^	−0.86	−1.186	NA	0.145	0.284	NA	−1.005	−1.47	NA
Cortet^12^	−0.7	NA	−1.1	−0.1	NA	−0.07	−0.6	NA	−1.03
Krajewska‐Wlodarczyk^17^	−1.28	−0.92	−1.09	−1.18	−0.94	−0.99	−0.1	0.02	−0.1
Kreeshan^18^	−1.4	NA	−1.2	−1.4	NA	−0.5	0	NA	−0.7
Nolla^24^	0.98	NA	0.81	0.97	NA	0.78	0.01	NA	0.03
Martinez‐Lopez^21^	−1.443	−0.63	NA	−0.098	−0.078	NA	−1.345	−0.552	NA
Modalsli^23^	0.07	0.02	0.05	−0.43	−0.44	−0.76	0.5	0.46	0.81
Riesco^27^	0.02	NA	−0.01	−0.33	NA	−0.16	0.35	NA	0.15
Solak^28^	−0.888	NA	−0.695	−0.412	NA	−0.49	−0.476	NA	−0.205

Abbreviations: FN, femoral neck; LS, lumbar spine; NA, not available; Ps, psoriasis; PsA, psoriatic arthritis; TH, total hip.

### 4.3 Ps treatment and OP/OPe

Medication use was recorded in 15 studies; specifically included TCS (*n* = 1), systemic corticosteroid (*n* = 8), NSAID (*n* = 3), DMARD (*n* = 10), Biologic (*n* = 7); anti‐osteoporotic (*n* = 2) and medications excluded (*n* = 4). Regarding the secondary outcome measure evaluating the possible effect of psoriatic medications on OP, nine studies performed subgroup analyses adjusting for medications. They were included for qualitative analysis, with three studies demonstrating a possible negative impact of systemic treatment on bone mineralization (Table 4).

**TABLE 4:** Psoriasis treatment and OP/OPe

Study	Included treatment	Other reported details	Medication sub‐analysis
Dreiher^1^	DMARD, biologic	Anti‐TNF 0.02%, IFN 0.1%, acitretin 0.1%, AZA 0.1%	Acitretin, anti‐TNF, IFN or AZA not associated with OP
Haddad^14^	SCS, NSAID, DMARD, biologic	NSAIDS, SCS (subgroup dispensing history: 0/1–2/≥3 prescriptions/year during study period), DMARD, biologic agent	Patients treated with DMARD higher estimated risk of OP 1.37 (95% CI 1.04–1.78)
Lee^19^	DMARD	Acitretin, SCS, CsA, MTX biologics excluded	No change with adjustment for DMARD use
Lo Giudice^20^	TCS, SCS, DMARD, biologics, anti‐osteoporotic	TCS >3 months (61%), ≥7.5 g prednisolone/equivalent daily >3 months (23%), bisphosphonate use (7%), DMARD (85%), biologics (30%)	SCS usage not associated with fragility fractures
Millard^22^	SCS, DMARD	SCS, acitretin, MTX	SCS, acitretin, MTX did not significantly affect BMD
Modalsli^23^	TCS, SCS, DMARD, biologic, HRT, anti‐osteoporotic	TCS (6%), SCS (3%), HRT (4%), anti‐osteoporotic therapy (3%); Dispensed prescribed drugs—Geralen, acitretin, fumaric acid, MTX, efalizumab, etanercept, adalimumab, CsA (infliximab not included)	No change with adjustment for SCS use
Petho^26^	SCS, DMARD, biologic	SCS (20%); DMARD – MTX, leflunomide, HCQ, sulfasalazine (45%); biologic – infliximab, adalimumab, etanercept, rituximab, abatacept, tocilizumab, certolizumab, golimumab, ustekinumab (53%)	Increased risk of hip fractures DMARD use
Riesco^27^	SCS, DMARD, biologic	SCS (37%), DMARD, anti‐TNF (20%)	No association between BMD and treatment with SCS, DMARD, anti‐TNFa
Zhu^29^	SCS, NSAID, DMARD, biologic	Nil	Vertebral fracture prevalence higher in SCS use (40 vs. 21.3%, *p* = 0.325)

Abbreviations: AZA, azathioprine; BMD, bone mineral density; CsA, cyclosporin; DMARD, disease modifying antirheumatic drug; IFN, interferon; MTX, methotrexate; NSAIDs, nonsteroidal anti‐inflammatory drugs; OP, osteoporosis; SCS, systemic corticosteroid; TCS, topical corticosteroid; TNF, tumour necrosis factor.

### 4.4 Reporting biases and certainty of the evidence

The risk of bias was moderate to high in most studies (Table 1). Inclusion and exclusion criteria meant that baseline characteristics of the psoriatic disease group were highly variable to begin, and the selection of non‐psoriatic controls was often poorly defined.

Overall, it is likely that there were too few studies in the metanalyses to draw definitive conclusions, but there were significant differences between Ps and control groups at face value. There was a very high level of heterogeneity with studies included in OR calculation (*I* ^2^ 99.7%; 95% CI 99.7–99.8; *Q* = 4876.06). Further influence analysis resulted in a non‐significant outcome; heterogeneity remained high even with excluding studies from analysis, and a formal test indicated publication bias.

In the meta‐analysis of areal BMD, omitting individual studies altered whether the difference in observed BMD was significant or not for LS and TH parameters. Heterogeneity was very high, particularly for LS, indicating substantial intra‐study difference and thereby questioning the validity of pooling data to obtain an overall effect. Further, there were not enough data to draw a definitive conclusion regarding publication bias, but visual inspection of relevant charts indicated that this was occurring.

## 5 DISCUSSION

The association between Ps and osteoporotic disease is contested. Ps has been purported to promote bone demineralization due to various mechanisms, including chronic systemic inflammation, the use of anti‐psoriatic medications and prolonged immobilization due to pain and joint dysfunction. This systematic review demonstrated a higher risk of OP/OPe with psoriatic disease by the odds ratio outcome and total BMD at the LS and FN, but not the TH. Furthermore, discrepancies were noted on qualitative analysis of *T*‐scores across different anatomical sites, and these variable findings are in keeping with other related systematic reviews.

Chen et al.,^30^ found no significant association between psoriatic disease and the absolute value of BMD. Further, patients with Ps did not have a higher risk of OP than the controls; but, interestingly, did have an increased OR of sustaining fractures unrelated to lower BMD/OP. Similar findings were reported by Sepehri et al.,^31^ demonstrating an increased risk of fracture but not OP/OPe in psoriatic disease. Chandran et al.,^32^ showed a possible association between various factors and low BMD in PsA, but with inconsistent findings. This reflects the complex relationship between Ps, PsA and low BMD and the multifactorial nature of potential risk factors related to Ps and its treatment.

There are many established risk factors for low BMD, and several included studies accounted for these confounders performing sub‐analyses. Females were found to have lower BMD^28^ and higher fracture risk^23^; conversely, another observed the association between Ps/OP observed only among males.^1^ Post‐menopause leads to lower BMD,^24^ increased fragility fractures^27^ and years since menopause determining BMD.^25^, ^26^ Vitamin D deficiency was associated with Ps^21^ and increased risk of hip fractures.^26^ Other studies have extrapolated that vitamin D deficiency plays a role in the pathogenesis of OP in psoriatic disease rather than psoriatic disease activity.^33^

Ps susceptibility genes are associated with genetic loci for metabolic syndrome, type 2 diabetes mellitus (DM), familial hyperlipidaemia, and cardiovascular disease.^25^ Studies included in this review found an overall higher prevalence of chronic disease comorbidities,^15^, ^19^, ^33^ with higher rates of endocrine (OR DM 1.56 and hypothyroidism 1.62)^1^, ^14^ and inflammatory conditions (rheumatoid arthritis, atopic dermatitis^19^). There was a higher risk of OP in Ps with obesity, but only in males^1^ and, interestingly, in one study, higher BMI was found to be protective with a higher *T*‐score.^21^

Heightened systemic inflammation levels in Ps represent a proposed mechanism for the association with low BMD, with higher C‐reactive protein (CRP) & erythrocyte sediment rate (ESR) in Ps.^28^ Although in the context of other evidence, there is a question of uncaptured cumulation of the effect of repeated attacks on bone demineralization.^33^ Other potential psoriatic disease‐specific risk factors for low BMD include higher PASI associated with lower *T*‐scores,^21^ the presence of erosions^11^ and an increased number of damaged joints.^13^ In addition, increased disease duration was associated with low BMD,^17^, ^26^ noting that one study did not find a significant correlation, albeit these groups were subdivided by an arbitrary less than or more than a 9‐year duration of arthritis.^13^ The presence of axial disease was excluded in two studies^24^, ^27^ but not explored elsewhere, with evidence suggesting more severe bone loss in peripheral PsA than axial PsA.^34^

Several Ps treatments have been shown to interfere with physiological bone metabolism, including corticosteroids,^35^ MTX,^36^ CsA.^37^ In Ps there is a dichotomy; AZA has been associated with overall increased fracture risk^38^ and glucocorticoids,^39^ CsA,^38^ and MTX^38^, ^40^ do not appear to impact bone health negatively. A large‐scale observational study, drawing on UK Biobank genomic data and using Mendelian randomization to evaluate the causal effect, concluded that the effect of PsA on OP was not genetically determined but secondary to treatment with medication (MTX, CsA).^41^ These results, however, must be interpreted with caution given methodological concerns, including high disease activity likely contributing to taking these medications in the first place.^42^, ^43^

In this review, most studies did not find any increased risk associated with Ps treatment; however, a few noted adverse outcomes associated with systemic corticosteroid^29^ and DMARD use.^14^, ^26^ As there was no standard approach to grouping medications across papers, and sub‐analysis for medications was only performed in a few‐studies, meta‐analysis was not performed. Biologics, as the newest therapeutic agents, were poorly represented, with at least one other study demonstrating no progression of catabolic and anabolic bone changes in the joints of patients with PsA with IL‐17 inhibition.^44^ Furthermore, emerging evidence suggests a beneficial effect of biologic treatment, that despite longer disease duration, there was better bone strength than patients receiving MTX or no DMARD,^45^ an area which perhaps warrants further attention.

Significant morbidity is associated with low BMD, with the degree of disability correlated to lower BMD.^13^ A large UK population‐based cohort study demonstrated that patients with Ps have an increased fracture incidence than the general population by up to 26%.^5^ This is in keeping with our findings, with an increase in fragility fracture associated with psoriatic disease^16^, ^26^, ^27^ bar one study.^20^ Other outcome measures such as increased hospitalization, loss of quality of life, economic burden and mortality were not within the scope of included studies.

Summarizing the results of studies within this systematic review demonstrates the considerable breadth of this subject. It perhaps explains the observed high level of heterogeneity, with individual studies focussing on different aspects of the complex interplay between Ps, PsA, OP and OPe. While most studies recorded additional population characteristics and comorbidities, they failed to adjust for these variables in subsequent analyses. Secondly, the methodological quality was highly heterogeneous, with potential selection bias introduced to both cases and controls and deficits in the length of follow‐up data. Notably, the largest study included^16^ (which if omitted renders the results not significant) encompassed event‐level records as opposed to patient‐level records. This means that the same patient could potentially be included more than once if they visited ED on multiple occasions. Further, several studies utilized national claim databases and diagnostic codes (i.e., ICD), introducing potential bias by coding errors and diagnosis misclassification. Finally, retrieved articles included brief reports, which scored poorly on the modified NOS quality assessment tool.

To our knowledge, this is the first systematic review evaluating BMD and psoriatic disease exclusively in studies with healthy control comparison groups and using outcomes of validated bone quality assessment measures. While these are strengths, this review is hampered by the small number of evaluable studies and demonstrates a reasonably high level of reporting bias. Given the small number of included studies, Ps and PsA groups were combined for analysis, due to variability of smaller groups and subsequent inability to perform useful subgroup analysis. As a result, we are unable to make a distinction between cutaneous Ps and PsA as a potential risk factor for low BMD, and there is a paucity of data related to cutaneous Ps alone. It is plausible that PsA alone may have a greater effect on BMD than Ps, given associated comorbidities and potential more frequent treatment with medications such as systemic corticosteroids. However this needs to be further evaluated in future specific studies.

The different modalities of expressing BMD (i.e., areal BMD as g/cm^2^, *T*‐score or *Z*‐score measured at multiple skeletal sites) diluted collected data further, making it difficult to draw meaningful conclusions. The gold standard of reporting BMD is using *T*‐scores, however due to the absence of studies reporting required variables, including means, standard deviations, medians, IQR, meta‐analysis was not performed. Chen et al.^30^ performed analyses using absolute BMD measurement only, and Chandran et al.^32^ also highlighted variable means of BMD reporting with subsequent limited analyses. Though it is the gold‐standard for OP diagnosis according to the World Health Organisation (WHO) classification, DXA has inherent flaws, with high specificity but low sensitivity, and may not encapsulate the actual risk of fracture in psoriatic disease.^30^, ^46^ Other outcome measures used to evaluate bone health and quality included FRAX, an assessment algorithm used in the prediction of hip fracture and other osteoporotic fractures, and quantitative computed tomography (QCT), a three‐dimensional quantification of volumetric BMD (g/cm^3^) and bone microstructure.^26^, ^29^ Notwithstanding, DXA remains the most widely validated tool for assessing BMD.

Finally, clearly accounting for medication use to allow sub‐analysis, or preferably designing a separate study evaluating specific individual treatment options would yield more results. Admittedly, this research topic is fraught with difficulty given polypharmacy and variation in personalized treatment regimens balanced against optimal disease control. Furthermore, disease severity and treatment are often closely interrelated, so it may be challenging to disentangle the role of chronic inflammation and systemic treatment on low BMD in severe Ps.

The limitations discussed highlight areas which require addressing in future studies to improve the quality of the literature. Further studies evaluating cutaneous psoriasis alone and the effect on OP are required, with studies being adequately powered to compare cutaneous psoriasis versus psoriatic arthritis, and OP. Importantly, standardized reporting methods of OP using *T*‐scores with additional parameters to allow use in meta‐analysis may yield more meaningful clinical conclusions. From the varied countries they were performed, the studies included had a diverse patient population. Ethnic background was sometimes specifically paired for controls^25^ but no sub‐analysis performed. Dheda et al.^13^ suggested Indians may be more at risk of PsA and low BMD than other Oriental ethnic groups, however further targeted studies may help identify if certain ethnic groups are at higher risk.

To reduce the burden of osteoporotic fracture, WHO advocates using clinical risk factors in conjunction with BMD to deliver timely interventions to individuals at high risk.^46^ While psoriatic disease as an independent risk factor of low BMD remains undetermined, using knowledge of established risk factors, patients with Ps generally have multiple and increased risk factors, placing them at higher risk of OP.^5^ This review highlights the need to be cognisant of the risk of OP in association with Ps and consider early evaluation and prophylactic treatment.

## 6 CONCLUSION

This systematic review evaluates BMD in psoriatic disease and treatment by summarising the published literature using healthy control comparison groups, synthesising existing evidence and highlighting deficits in current knowledge. It shows inconsistencies and a likelihood of bias in the published literature about low BMD prevalence and medication risk factors in Ps and PsA. This study highlights that Ps patients should be monitored for complex comorbidity, including OPe/OP. However, more extensive, longitudinal studies or Ps registries, including robust assessment of comorbidities and pharmacological treatment, together with more standardised BMD reporting measures, are needed to more accurately characterise the risk of low BMD in cutaneous Ps.

## CONFLICT OF INTEREST

None to declare.

## AUTHOR CONTRIBUTIONS

**Anna Schauer**: Conceptualization (Lead); Formal analysis (Lead); Project administration (Equal); Writing – original draft (Lead); Writing – review & editing (Equal). **Aarthy K. Uthayakumar**: Conceptualization (Supporting); Formal analysis (Supporting); Project administration (Equal); Writing – review & editing (Equal). **Glenn Boardman**: Formal analysis (Lead); Methodology (Lead). **Christopher B. Bunker**: Conceptualization (Supporting); Supervision (Lead); Writing – review & editing (Supporting).

## ETHICS STATEMENT

Not applicable.

## Supporting information

Supporting Information S1

## Data Availability

Data sharing is not applicable to this article as no new data were created or analyzed in this study.
