# Supplementary material for: The risk of osteopenia/osteoporosis and psoriatic disease: A systematic review
Source: Skin Health Dis. 2022 Sep 21;3(1):e169. doi: 10.1002/ski2.169 (PMC9892432; doi:10.1002/ski2.169)
Supplement: Supplementary file 1 — Supporting Information S1 [file SKI2-3-e169-s001.docx]

**Supporting information**

**Table S1**. Ovid (Embase) search strategy and results

| # | searches | results |
| --- | --- | --- |
| 1 | exp psoriasis | 91840 |
| 2 | Osteoporosis.m_titl. | 38687 |
| 3 | Osteopenia.m_titl. | 2408 |
| 4 | Fracture.m_titl. | 71192 |
| 5 | Bone.m_titl. | 313951 |
| 6 | 2 or 3 or 4 or 5 | 410463 |
| 7 | 1 and 6 | 632 |
| 8 | Limit 7 to (human and English language) | 528 |
| 9 | clinical study/ | 156839 |
| 10 | exp case control study/ | 187639 |
| 11 | longitudinal study/ | 152459 |
| 12 | Retrospective study/ | 1045164 |
| 13 | Prospective study/ | 670534 |
| 14 | Cross-sectional study/ | 398558 |
| 15 | Cohort analysis/ | 682025 |
| 16 | Follow-up/ | 1659845 |
| 17 | Cohort*.ti,ab. | 1101490 |
| 18 | 16 AND 17 | 247034 |
| 19 | case control.ti,ab. | 173038 |
| 20 | (cohort adj (study or studies or analys*)).ti,ab. | 346674 |
| 21 | ((follow up or observational or uncontrolled or non randomi#ed or nonrandomi#ed or epidemiologic*) adj (study or studies)).ti,ab. | 370054 |
| 22 | ((longitudinal or retrospective or prospective or cross sectional) and (study or studies or review or analys* or cohort*)).ti,ab. | 2346231 |
| 23 | 9 OR 10 OR 11 OR 12 OR 13 OR 14 OR 15 OR 18 OR 19 OR 20 OR 21 OR 22 | 3889625 |
| 24 | 8 AND 23 | 127 |

**Table S2**. PUBMED search strategy and results

(“Psoriasis”[Mesh] AND (“Osteoporosis”[Mesh] OR osteoporosis[tw] OR osteopenia[tw])

(Psoriasis[MeSH Major Topic]) AND (Osteoporosis[MeSH Major Topic])

| # | searches | results |
| --- | --- | --- |
| 1 | (Psoriasis[MeSH Major Topic]) AND (Osteoporosis[MeSH Major Topic]) | 44 |
| 2 | Limited to English humans | 38 |

**Table S3.** Modified Newcastle-Ottawa Scale for case control studies (maximum: 9 stars)^^^

| **Selection** |
| --- |
| 1. Representativeness of the exposed cohort (psoriatic disease) |
| - 1. truly or somewhat representative of the general population of psoriatic disease * |
| - 1. potential for selection bias or not stated |
| 1. Selection of non-psoriatic controls |
| - 1. drawn from the same community as the exposed cohort * |
| - 1. drawn from a different source |
| - 1. no description of the derivation of the non-exposed cohort |
| 1. Ascertainment of psoriatic disease (maximum one *) |
| - 1. secure records (eg. hospital records) * |
| - 1. International Classification of Diseases (ICD) code* |
| - 1. self-reported |
| - 1. no description |
| 1. Demonstration that outcome of interest was not present at start of study |
| - 1. yes * |
| - 1. no |
| **Comparability** |
| 1. Comparability of the exposed group and the non-psoriatic controls on the basis of the design or analysis |
| - 1. study controls for age* |
| - 1. study controls for any additional factor* |
| - 1. no description |
| **Outcome** |
| 1. Assessment of outcome (maximum one *) |
| - 1. secure record (records of bone mineral density measurement, osteoporosis) * |
| - 1. structured interview * |
| - 1. self-report only |
| - 1. no description |
| 1. Same method of ascertainment for cases and controls |
| - 1. yes * |
| - 1. no |
| 1. Non-response rate |
| - 1. same response for both groups * |
| - 1. non-respondents described |
| - 1. no description |

^A study may be awarded a maximum of one star for each numbered item for the Selection and Exposure categories and maximum of two stars for Comparability

**Table S4.** Modified Newcastle-Ottawa Scale for cohort studies (maximum: 9 stars)^^^

| **Selection** |
| --- |
| 1. Representativeness of the exposed cohort (psoriatic disease) |
| - 1. truly or somewhat representative of the general population of psoriatic disease * |
| - 1. potential for selection bias or not stated |
| 1. Selection of non-psoriatic controls |
| - 1. drawn from the same community as the exposed cohort * |
| - 1. drawn from a different source |
| - 1. no description of the derivation of the non-exposed cohort |
| 1. Ascertainment of psoriatic disease (maximum one *) |
| - 1. secure records (e.g. hospital records) * |
| - 1. International Classification of Diseases (ICD) code* |
| - 1. self-reported |
| - 1. no description |
| 1. Demonstration that outcome of interest was not present at start of study |
| - 1. yes * |
| - 1. no |
| **Comparability** |
| 1. Comparability of the exposed group and the non-psoriatic controls on the basis of the design or analysis |
| - 1. study controls for age* |
| - 1. study controls for any additional factor* |
| - 1. no description |
| **Outcome** |
| 1. Assessment of outcome (maximum one *) |
| - 1. secure record (records of bone mineral density measurement, osteoporosis) * |
| - 1. structured interview * |
| - 1. self-report only |
| - 1. no description |
| 1. Was follow-up long enough for outcome |
| - 1. yes ≥5 years* |
| - 1. no ≤5 years |
| 1. Adequacy of follow up of cohorts (maximum one *) |
| - 1. complete follow up-all subjects accounted * |
| - 1. subjects lost to follow up unlikely to introduce bias (loss of follow-up rate <20%) * |
| - 1. loss of follow-up rate >80% |
| - 1. no description of the lost |

^A study may be awarded a maximum of one star for each numbered item for the Selection and Exposure categories and maximum of two stars for Comparability

**Table S5.** Modified Newcastle-Ottawa Scale for cross-sectional studies (maximum: 6 stars)^^^

| **Selection** |
| --- |
| 1. Representativeness of the exposed cohort (psoriatic disease) |
| - 1. truly or somewhat representative of the general population of psoriatic disease * |
| - 1. potential for selection bias or not stated |
| 1. Selection of non-psoriatic controls |
| - 1. drawn from the same community as the exposed cohort * |
| - 1. drawn from a different source |
| - 1. no description of the derivation of the non-exposed cohort |
| 1. Ascertainment of psoriatic disease (maximum one *) |
| - 1. secure records (e.g. hospital records) * |
| - 1. International Classification of Diseases (ICD) code* |
| - 1. self-reported |
| - 1. no description |
| **Comparability** |
| 1. Comparability of the exposed group and the non-psoriatic controls based on the design or analysis |
| - 1. study controls for age* |
| - 1. study controls for any additional factor* |
| - 1. no description |
| **Outcome** |
| 1. Assessment of outcome (maximum one *) |
| - 1. secure record (records of bone mineral density measurement, osteoporosis) * |
| - 1. structured interview * |
| - 1. self-report only |
| - 1. no description |

^A study may be awarded a maximum of one star for each numbered item for the Selection and Exposure categories and maximum of two stars for Comparability


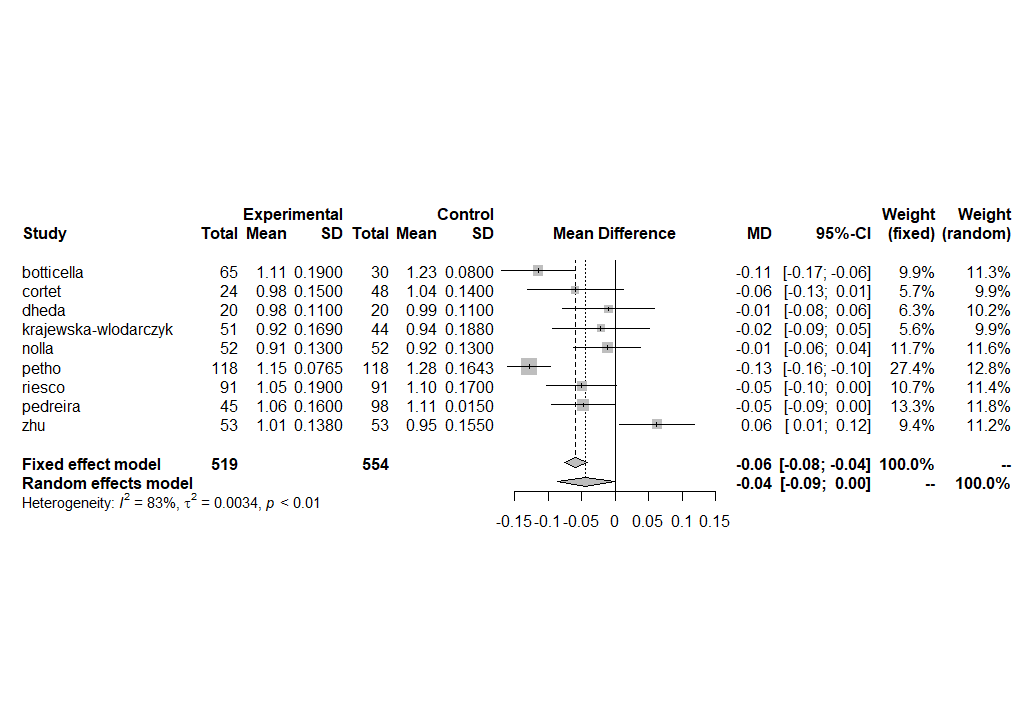


**Figure S1.** Pooled association between Lumbar Spine Bone Mineral Density and Psoriatic disease


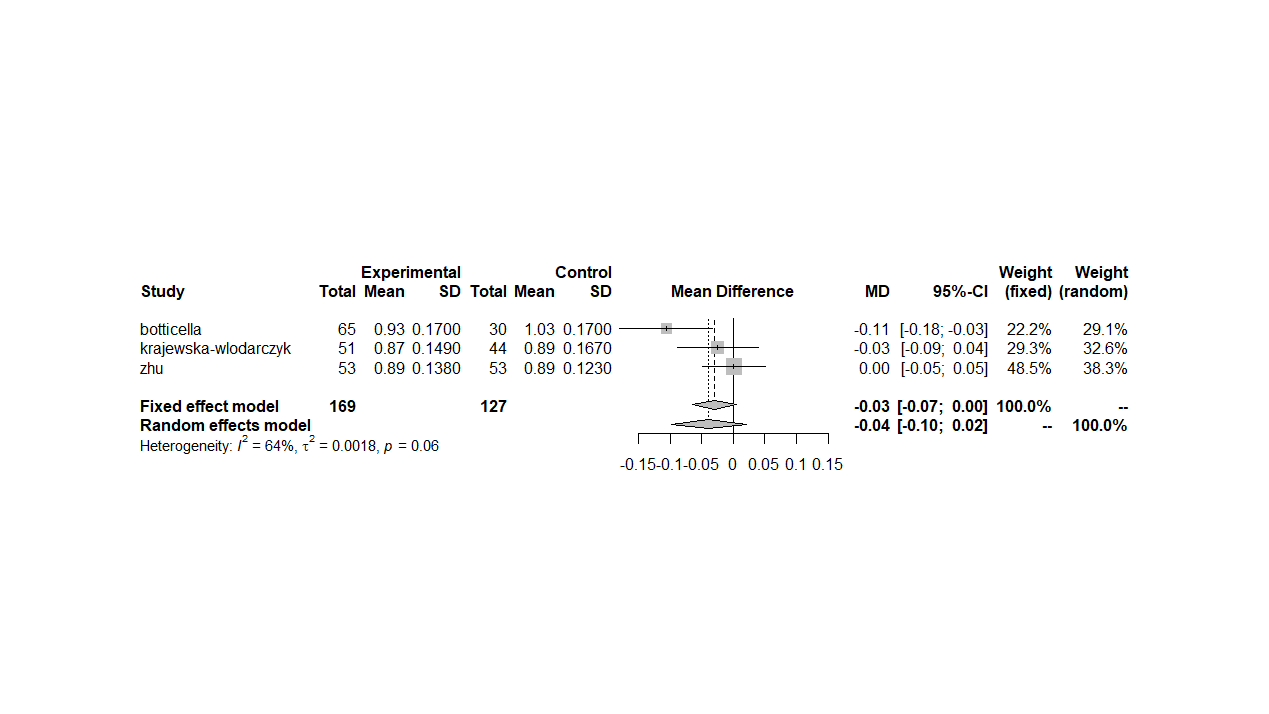


**Figure S2.** Pooled association between Total Hip Bone Mineral Density and Psoriatic disease


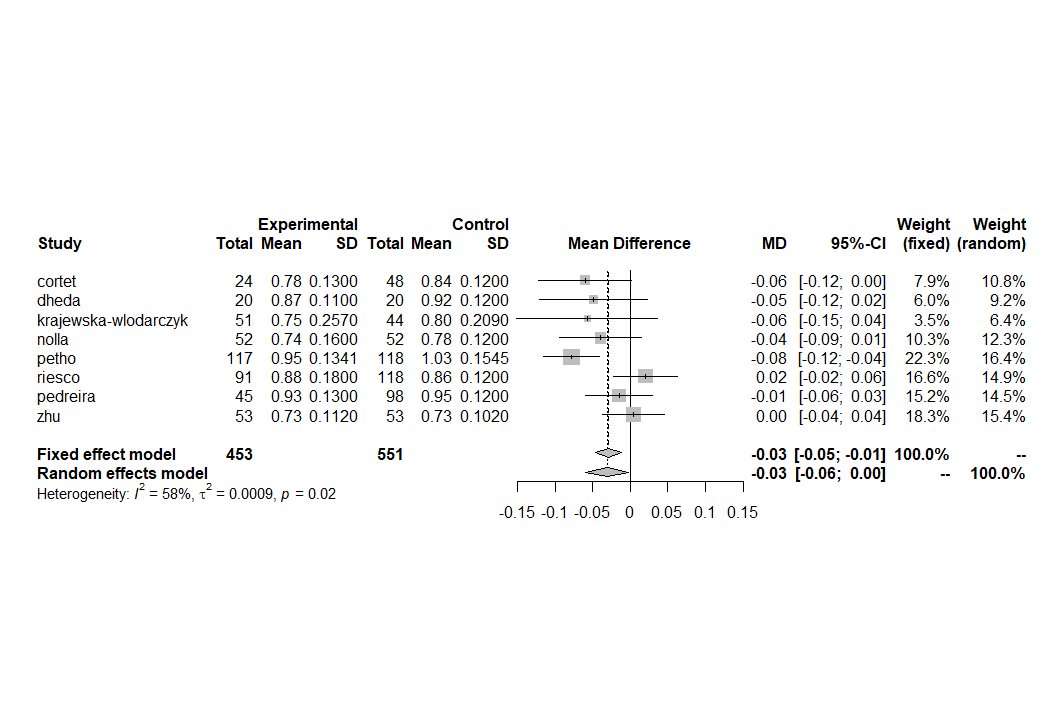


**Figure S3.** Pooled association between Femoral Neck Bone Mineral Density and Psoriatic disease
